# Differential venous oxygen return: a key factor of differential hypoxia in venoarterial extracorporeal membrane oxygenation

**DOI:** 10.1186/cc14358

**Published:** 2015-03-16

**Authors:** X Hou, X Yang, Z Du, J Xing, C Jiang, J Wang, Z Xing, H Wang, H Zeng

**Affiliations:** 1 Beijing Anzhen Hospital, Beijing, China; 2 Beijing Key Laboratory of Emerging Infectious Diseases, Beijing, China

## Introduction

Differential hypoxia is a pivotal problem in cardiopulmonary failure patients with femoral venoarterial extracorporeal membrane oxygenation (VA ECMO) support. Although there was some attempt to deliver more oxygenated blood to the upper body, the mechanism of differential hypoxia has not been well investigated.

## Methods

We used a sheep model of acute respiratory failure that was supported with femoral VA ECMO (from inferior vena cava to femoral artery (IVC-FA)), ECMO from superior vena cava to FA (SVC-FA), ECMO from IVC to carotid artery (IVC-CA) and ECMO adding an additional return cannula to internal jugular vein based on femoral VA ECMO (FAIJV). Angiography and blood gas analysis were performed.

## Results

**Figure 1:**
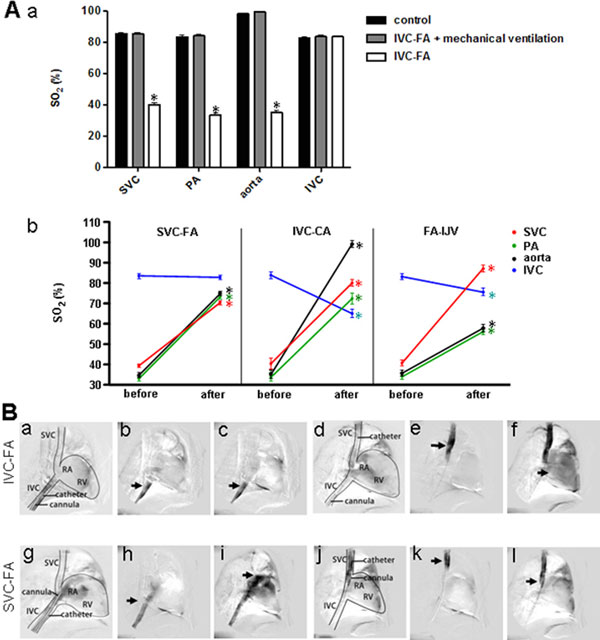
**SO_2_ values from blood gas analysis and vena cava angiography**.

Blood oxygen saturation (SO_2_) of IVC (83.6 ± 0.8%) was higher than that of SVC (40.3 ± 1.0%) in sheep with IVC-FA. Oxygen-rich blood was drained back to the ECMO circuit and poorly oxygenated blood in the SVC entered the right atrium (RA). SVC-FA achieved the oxygen-rich blood return from IVC to RA without shifting the arterial cannulation. SO_2_ of SVC and pulmonary artery increased (70.4 ± 1.0% and 73.4 ± 1.1%, respectively) subsequently. Compared with IVC-FA, less differences of venous oxygen return and attenuated differential hypoxia were also observed in IVC-CA and FA-IJV. See Figure 1.

## Conclusion

Differential venous oxygen return is a key factor of differential hypoxia in VA ECMO. We can take advantage of the notion of differential venous oxygen return to choose better cannula configuration in clinical practice.

